# Chronic Obstructive Pulmonary Disease and Overtraining Syndrome: A Narrative Review

**DOI:** 10.7759/cureus.105016

**Published:** 2026-03-10

**Authors:** Bruno Bordoni, Enricomaria Mattia, Bruno Morabito

**Affiliations:** 1 Department of Physical Medicine and Rehabilitation, Fondazione Don Carlo Gnocchi, Milan, ITA; 2 Department of Physical Medicine and Rehabilitation, Centro di Radiologia Medica e Terapia Fisica Morrone, Servizio Sanitario Nazionale, Caserta, ITA; 3 Faculty of Medicine, Università Cattolica del Sacro Cuore, Agostino Gemelli University Hospital, Rome, ITA

**Keywords:** american thoracic society, copd, diaphragm, european respiratory society, fev1, inspiratory muscle training, maximal inspiratory pressure, overtraining syndrome, rehabilitation training

## Abstract

Chronic obstructive pulmonary disease (COPD) is a condition that inevitably leads to airflow limitation. COPD is among the leading causes of increased mortality and morbidity worldwide. A non-invasive and non-pharmacological approach is rehabilitation training, where the patient follows an active program to stimulate the limb and respiratory muscles. Training involves a constant increase in workloads throughout the rehabilitation process. A fundamental concept absent from the literature is that of including training sessions with reduced loads and periods of "unloading" intensity within the rehabilitation program. Without adequate recovery and rest between sessions, the patient may lack the resources necessary to tackle a subsequent demanding rehabilitation session. This situation could lead to the onset of overtraining syndrome (OTS), where the patient experiences an unexplained decline in performance. The article reviews the muscular adaptation of COPD patients and the planned rehabilitation and emphasizes the concept that clinicians should structure the rehabilitation training program not in a linear fashion (constantly increasing loads), but in a wave-like fashion (scheduling some sessions with decreased loads). This organization could benefit the patient's performance, reducing the risk of OTS.

## Introduction and background

Chronic obstructive pulmonary disease (COPD) is a progressive limitation of airflow, with alveolar structural alterations and symptoms that chronically limit the quality of life of patients. The prevalence of COPD worldwide is variable, and it may represent the third most common cause of disease in China and the sixth most common cause of disease in the United States [1,2]. Globally, COPD is the third leading cause of death, with approximately three million deaths worldwide [3]. The severity classification parameters of the patient according to the Global Initiative for Chronic Obstructive Lung Disease (GOLD) can be classified based on the value derived from the post-bronchodilator forced expiratory volume in one second (FEV1): mild (FEV1 ≥80% of predicted), moderate (FEV1 50-79% of predicted), severe (FEV1 30-49% of predicted), and very severe (FEV1 <30% of predicted) [1]. We can find a fifth stage or GOLD 0 (pre-COPD), in which patients with smoking (or non-smokers) present increased airway wall thickness. The latter have a high percentage of probability of developing COPD [4].

A new proposal for classifying COPD severity is the STaging of Airflow obstruction by Ratio (STAR). It is based on the FEV1/forced vital capacity (FVC) ratio values: stage 4 (<0.4), stage 3 (≥0.4-0.5), stage 2 (≥0.5-0.6), and stage 1 (≥0.6-0.7) [1]. This latter classification may be more sensitive to the severity of the clinical picture and mortality [4].

Acute exacerbation of chronic obstructive pulmonary disease (AECOPD) is common, increasing the mortality rate one year after the acute event by 26.2% and by 64.3% five years after the exacerbation [4].

Symptoms and concomitant pathologies/negative related effects are variable, as are the underlying causes of the disease. Patients with COPD may complain of dyspnea, cough and phlegm, fatigue, and a constellations of dysfunctions that are not always easy to explain or connect to the GOLD classification: insomnia, dry mouth, drowsiness, chest and/or diffuse pain, anorexia, intestinal and gastric disorders, nausea, wheezing, sleep apnea, dysphagia, urinary incontinence, temporomandibular disorders and/or Costen's syndrome, anxiety and depression, recurrent falls and sarcopenia, tiredness, lung cancer, heart problems, osteoporosis, psoriasis, stroke, nonalcoholic fatty liver disease, polyneuropathy, chronic kidney disease, diabetes, malnutrition, reduced neurocognitive function, and visual dysfunctions [5-13].

The article reviews the muscular adaptation that COPD patients undergo and highlights the concept of absence in the guidelines and research of overtraining syndrome (OTS), where failure to respect periods of "unloaded" work poses the risk of an inadequate response to rehabilitation, with the onset of a plethora of symptoms that are not always easy to define. The narrative review searched PubMed for the most recent research on muscle adaptation in COPD patients and rehabilitation training guidelines. Finally, the most relevant texts on the concept of OTS, its possible causes, and its related symptoms were searched (literature from 2000 to 2026, with exceptions for one article from 1996 and one article from 1998). OTS is a non-physiological systemic adaptation. We recall that the concept of OTS in the clinic with patients with COPD has not yet been demonstrated and further studies will be needed to fill this gap.

## Review

The causes that induce chronic pathological adaptation to the airways are pollution, smoking, the occurrence of asthma in childhood, prematurity, various airway infections during growth, and genetic alteration (alpha-1 antitrypsin deficiency). Approximately 50% of the causes of COPD are represented by pollution (ozone, nitrogen oxides, black carbon, and fine particulate matter PM2.5) [14]. Air pollution stimulates tissue oxidation and chronic inflammatory responses of airway epithelial cells and immune cells, respectively [14]. Tobacco (approximately 1.3 billion smokers worldwide) is another cause that stimulates systemic inflammation and oxidation, with endothelial alteration (increased arterial stiffness and thickness), which contributes to the onset of COPD [15].

**Figure 1:**
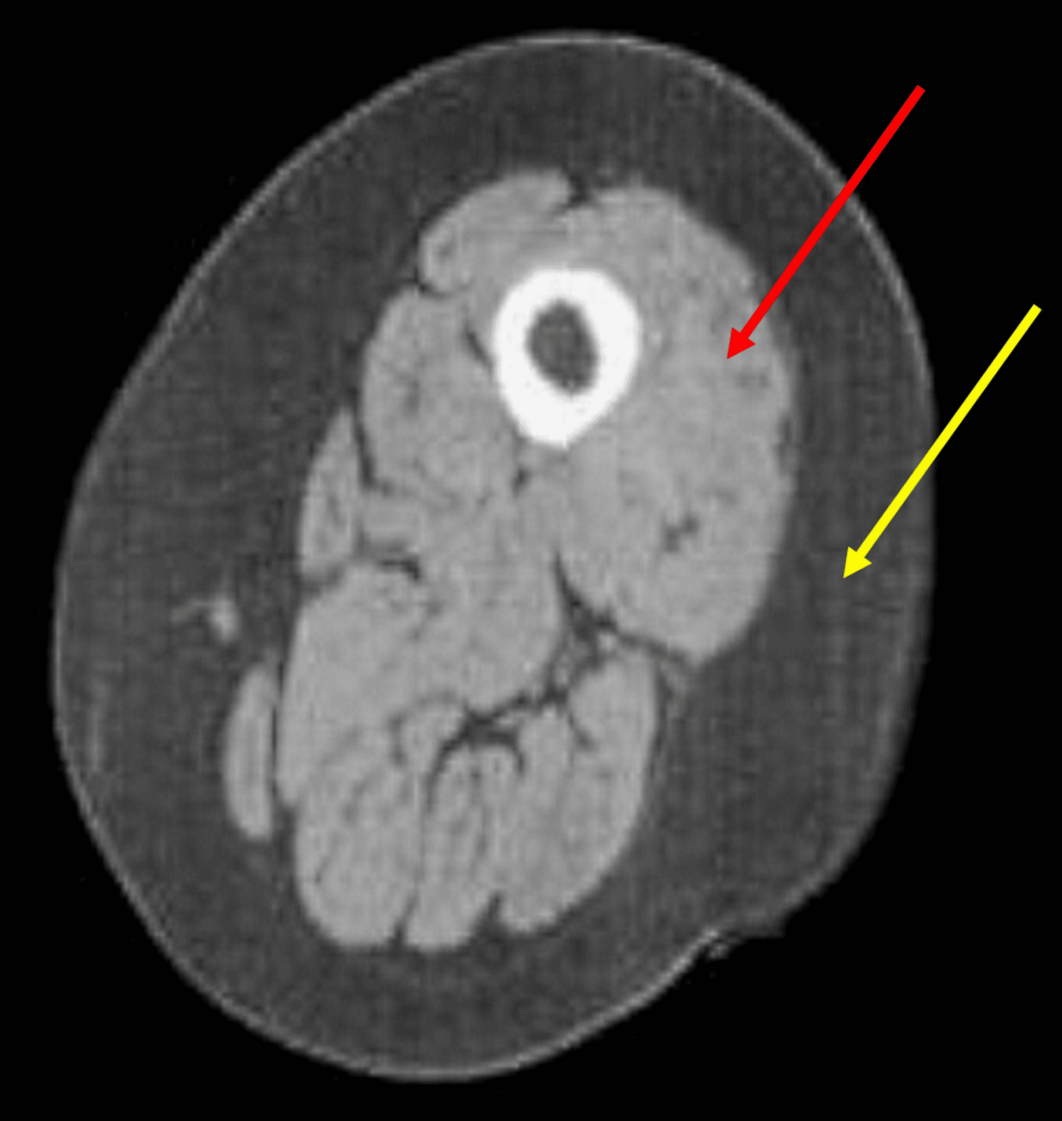
MRI shows sarcopenia of the thigh area of ​​a patient with COPD (with depressed FEV1, 50-79% of predicted). Hypotrophy (light gray area, red arrow) can be noted, with the presence of subcutaneous and intramuscular fat (dark gray area, yellow arrow) MRI: magnetic resonance imaging; COPD: chronic obstructive pulmonary disease; FEV1: forced expiratory volume in one second Image Credit: Bruno Bordoni

A condition that is found in all patients with COPD is muscle dysfunction (functional, morphological, and phenotypic alterations), where skeletal muscles undergo a worsening adaptation as the disease worsens and become a prognosis for the evolution of the respiratory pathology [16]. Patients with emphysema undergo a greater negative adaptation at the muscular level; patients with a moderate degree of obstruction may present a high level of sarcopenia that does not necessarily correlate with spirometric parameters (Figure 1).

The lower limbs undergo a greater pathological adaptation than the upper limbs, with a greater decrease in function and volume in females than in males [5]. In both genders, a phenotypic shift is observed, with a decrease in aerobic fibers (myosin heavy chain (MyHC) type I), rarefaction of the capillary scaffold, mitochondrial and ribosomal dysfunction, and an increase in anaerobic fibers MyHC type II). Women have a greater number of hybrid fibers, highlighting a further difficulty in metabolic and regenerative adaptation [5]. The sensitivity of troponin C to calcium is decreased, as is the ability to reuptake calcium from the sarcoplasmic reticulum; the muscle is stiffer and less able to manage tone [17]. An alteration in the synthesis of titin is found, making the fiber more fragile [17].

Frequent acute rehospitalizations (AECOPD) accelerate the loss of strength and muscle mass; after five days of hospitalization, muscle strength is lost approximately 5%, compared to non-hospitalized COPD patients [5]. This pathological adaptation worsens the prognosis [5]. Electromyography is altered, revealing neuromuscular incoordination and rapid fatigue, with an increased mortality rate [5].

It is not easy to identify a single cause leading to loss of muscle mass with metabolic alterations, as multiple factors and different sarcopenic pathways contribute: smoking, hypoxia, a sedentary lifestyle, hypercapnia, recurrent infections, corticosteroid drugs, malnutrition, a decrease in anabolic hormones, a decrease in regenerative capacity (fewer satellite cells), intracellular calcium toxicity, oxidative stress, and the presence of the ubiquitin-proteasome and lysosomal-autophagy pathways [17].

Pathological adaptation of the diaphragm

**Figure 2:**
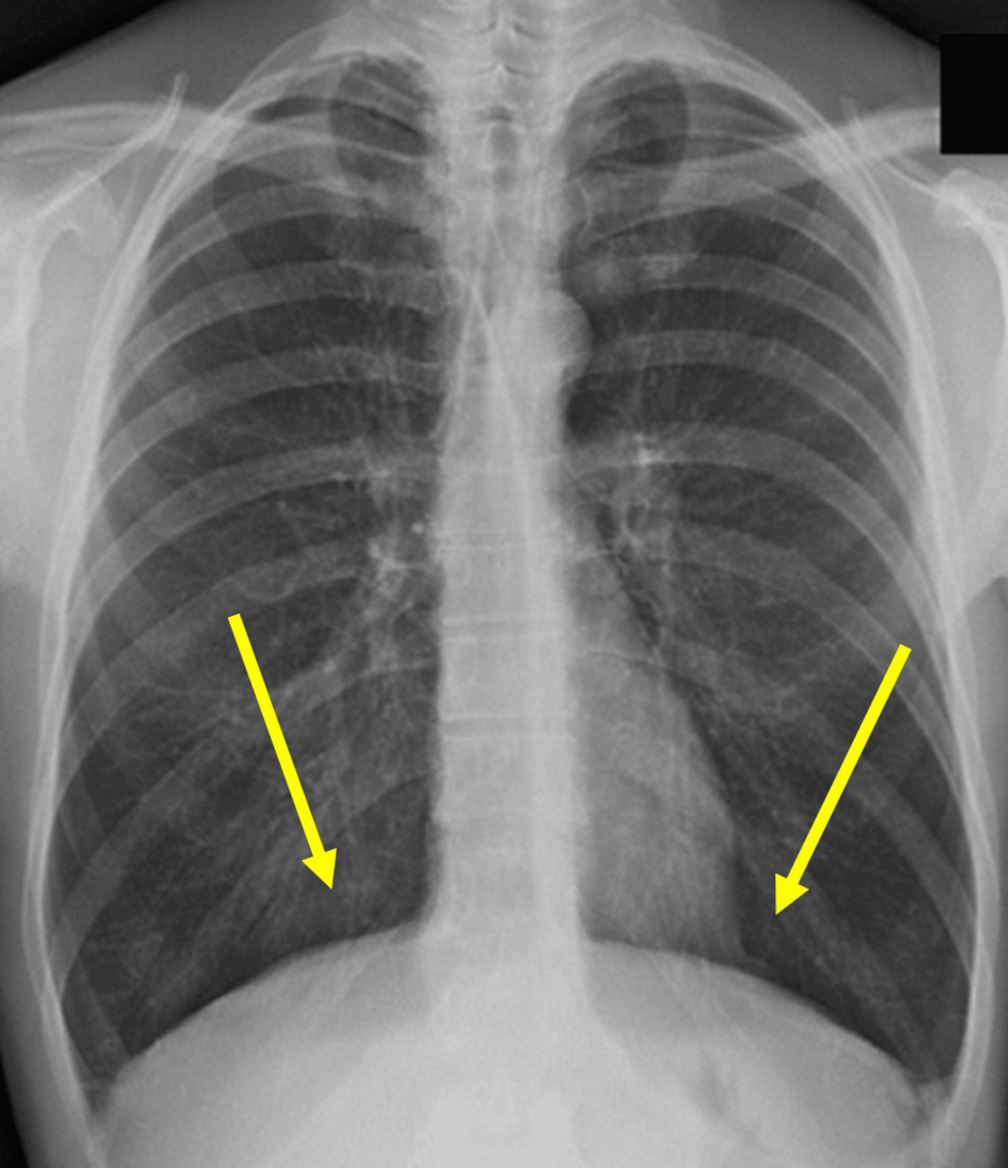
Chest X-ray of a COPD patient. The "rounded" morphology (arrows) of the diaphragm is noted, which is in an inspiratory posture, with shortened fibers COPD: chronic obstructive pulmonary disease Image Credit: Bruno Bordoni

Another extraordinarily important muscle for proper lung function is the diaphragm. Patients with COPD have lower transdiaphragmatic pressure (Pdi) and maximal inspiratory pressure (PImax) values ​​than healthy subjects [5]. The diaphragm appears in an inspiratory posture, with chronically shortened fibers (by approximately 28%); its morphology is altered, with a "rounded" diaphragm [5]. This morphology reflects the presence of hyperinflation and dyspnea (Figure 2).

The inspiratory attitude decreases the area of ​​apposition of the diaphragm (with less leverage for contraction) and increases the radius of curvature (decreases the tone produced in the unit of time); the excursion is reduced with a markedly lower expression of force [5]. A phenotypic change occurs, where aerobic fibers increase and anaerobic fibers decrease, with a timing of phenotypic shift that is faster than the contractile change of the limbs [5]. The diaphragmatic muscle tissue undergoes atrophy (loss of myosin content of approximately 50% and decreased quantity of nebulin), fibrosis, sclerosis, and myolysis; the sarcoplasmic reticulum is dysfunctional, with increases in calcium cytotoxicity, which will cause apoptotic responses of the cell and a further decreased contractile capacity (decreased capacity to form actomyosin bridges). The force expressed by the diaphragm is approximately 35% lower than in healthy subjects [5].

Titin does not appear to be reduced in quantity, but its shock-absorbing capacity is altered, making the fiber more fragile; there is a misalignment as well as a loss (approximately 10-15%) in the number of sarcomeres [5]. The FEV1 value is directly linked to the degree of diaphragmatic hypotrophy [5].

The dyspnea complained of by the patient is not directly related to pulmonary deterioration, but to the muscular strength of the diaphragm; furthermore, the thoracic muscles become thinner over time and less effective at expanding the lungs [5]. The phrenic nerve slows electrical conduction with myelin damage (typical of neuropathy); the left phrenic nerve appears to suffer greater neuropathic damage, but the reasons are unclear. The pathological adaptation of the phrenic nerve is directly linked to FEV1 values ​​[5]. The capillary scaffold is preserved, despite a reduction in endurance capacity.

The weakness expressed by the diaphragm not only is a prognosis of the disease but also correlates with the percentage of hospitalization/AECOPD [5].

Rehabilitation training

One approach that can slow the decline of skeletal and respiratory muscles is rehabilitation training for patients with COPD [18-21]. The goal of training with weights and machines with sets and repetitions (strength/resistance training), and with tools such as exercise bikes and treadmills (endurance training, high-intensity interval training), is to improve the patient's performance or reduce their decline for an acceptable daily autonomy [18-21]. We could compare the patient to an athlete, since, although the motivations are different, the objectives are the same: improving performance. In European, American, and Asian guidelines, for endurance training, values ​​are applied that reflect 60-80% of the maximum subjective aerobic work capacity (values ​​derived from spirometry or exercise test with oxygen consumption) and over 80% for high-intensity interval training; the duration varies from 20 minutes to an hour, with weekly sessions of 3-5 days [18-25]. As regards resistance training, the usual choice is to use loads of 60-80% of the subjective maximum contractile capacity or one repetition maximum with a specific weight (1RM), with 2-5 sessions per week, 8-12 repetitions, and 2-4 sets for the major muscle groups [18-24].

For respiratory muscles such as the diaphragm (inspiratory muscle training (IMT)), not all international guidelines provide specific suggestions for IMT, despite local muscular and systemic improvements (maximum excursion of the diaphragm, peak oxygen consumption, PImax, Pdi) [5,25]. Compared to the current literature, there is a tendency to apply inspiratory resistance with specific instrumentation that reflects 9-133% of the subjective PImax, with a training duration of 5-60 minutes, and 2-15 sets, with 2-7 sessions per week and a variable number of breaths [26-29].

The guidelines always provide for an increase in workload when the patient reports less difficulty completing the rehabilitation session.

Areas for improvement

What is surprisingly missing is the consideration that, as for any athlete undergoing training, the patient must also follow a training program that includes a cool-down period (days or weeks). In this phase, the patient/athlete has the possibility of obtaining a recovery period from previous training sessions, thus improving performance (supercompensation) [30]. No athlete during the training period (monthly or yearly) always increases the workloads, as happens, instead, in the rehabilitation program of patients with COPD (intensity is increased by 5-30% over the initial load at variable intervals) [18-24,26-30]. In the cool-down phase, the training loads are lower than those used in the load increase phase. If a cool-down is not respected in which the training loads are not increased and/or the intensity is not lowered, the risk is that of falling into OTS [31,32]. Not only does performance tend to decline, but cognitive and mood aspects also worsen [31,32]. Rehabilitation training should be seen not as a straight line going uphill, but as a wavy line going upward.

**Figure 3:**
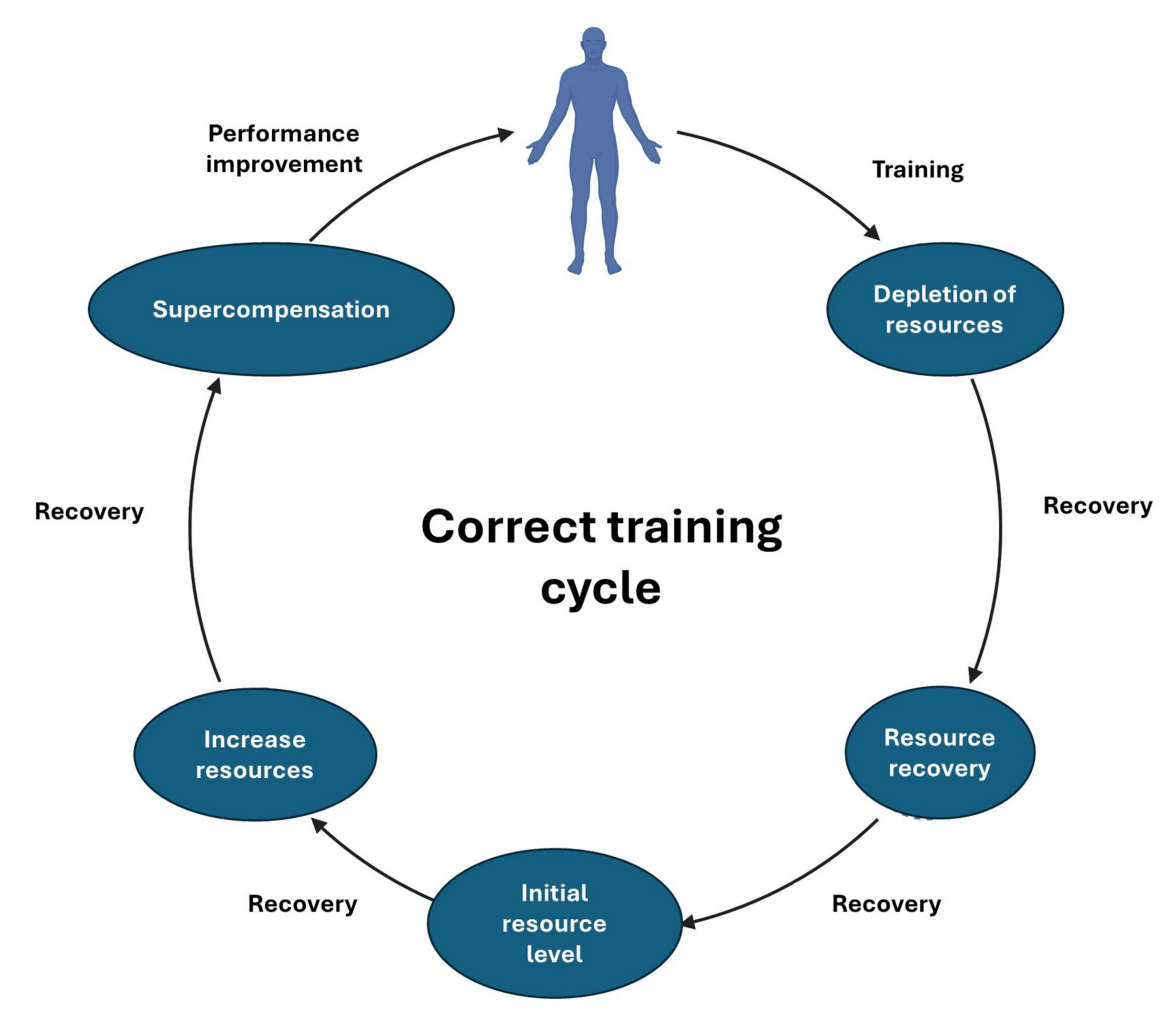
The image illustrates how proper training allows for improved performance; the training stimulus depletes the body's resources, and only with adequate rest can the depleted resources be replenished to achieve supercompensation, that is, resources beyond those initially available during training to better withstand the recurrence of the training stimulus, thus improving performance Image Credit: Bruno Bordoni, created in BioRender

After a rehabilitation session, the body's resources have been used, and only during the recovery/rest period are they recovered; if the expected recovery time is optimal, the body adds further resources, or supercompensation. This mechanism allows not only to improve performance but also to address the same training stressors with fewer resources used by the body [33]. It is during rest that the body can improve, not during training. A different culture is needed in the clinical and research fields, where this simple but incredibly under-implemented concept is envisioned. We do not know what happens to the patient if a cool-down period is inserted into the rehabilitation process. It is possible to hypothesize that the performance and psycho-physical well-being results could be improved (Figure 3).

Research is a navigation; deciding which route to take will determine the precise point at which one can arrive.

Linear and non-linear periodization of rehabilitation

These models originally date back to the 1970s and 1980s for the preparation of weightlifters for Olympic competitions, where only one competition per year was scheduled [34]. The linear model (LP) considers the year as a macrocycle, the months as a mesocycle (2-3 months), and a microcycle of 1-4 weeks. LP involves increasing workload over the course of the different mesocycles, without providing for unloading periods; many rehabilitation protocols apply this concept [34,35]. Each microcycle sees an increase in workload resistance [35].

The "advantage" of following LP is that of not planning different schemes depending on the pathology, but on the clinical state of the patient. The objective of the training is to increase fatigue during the rehabilitation process. The non-linear model (non-LP) consistently predicts a steady increase in workload, provided that the patient does not experience the same level of fatigue as in the preceding rehabilitation session [35].

Considering that a rehabilitation program lasts an average of a couple of months for patients with COPD, workloads can increase during this time without noticing symptoms of performance decline. However, patients are not taught how to adequately plan their own home rehabilitation, as their resources are never infinite. LP and non-LP make rehabilitation planning much easier for the therapist [34].

The disadvantage is creating an environment where central and peripheral neurological systems are unable to regenerate the energy used during rehabilitation sessions, causing OTS [35].

Reverse linear periodization (RLP)

In this model (RLP), an increase in work volume (session time and rest between exercises) is expected over the mesocycle, but with a decrease in loads to be overcome (speed, resistance) [35]. There are no examples in the literature involving RLP and patients with COPD. The concept, we reiterate, is not to create fatigue in the patient simply by changing training variables, but to create a work environment that allows the individual to achieve optimal recovery from the training stimulus, thereby improving performance and avoiding OTS. Without comprehensive recovery throughout the rehabilitation training, the patient is at risk of experiencing OTS, just like a healthy, athletic individual [36].

OTS

How can you tell if a patient is in OTS? The reasons are not always clear and exhaustive [37]. When a person is unable to sustain the load of previous rehabilitation training, without apparent justification (stable clinical values ​​and unchanged instrumental tests), and this event is repeated in subsequent sessions, overtraining has most likely set in [37,38]. One of the reasons is a disruption of the neuroendocrine-immunological system without appropriate rest, which could cause a cascade of systemic inflammatory processes and mood changes [37,38]. The associated symptoms are multiple and not easy to classify (Table 1).

**Table 1:** Main symptoms associated with overtraining syndrome that can be found in healthy athletes

General symptoms of overtraining syndrome
Alterations in blood pressure and heart rate
Chronic fatigue
Burnout
Stress fractures
Sleep disturbances
Changes in body weight
Easily distractible
Mood changes (deflection or agitation)
Chronic muscle pain

There are no gold standard clinical tests for the detection of OTS, and there is no gold standard treatment [37]. When OTS occurs, weeks or months are needed before a complete recovery of bodily functions [37]. Prevention (adequate rest during rehabilitation sessions and correct management of workloads) probably remains the most effective gold standard to avoid the onset of this chronic alteration [36,37,39]. Naturally, subjective elements must be taken into consideration, such as the nutritional properties consumed or lacking, the social and working environment, previous pathologies and pharmacology taken, previous traumas, family history, and so on. Subjectivizing rehabilitation training plans is essential to prevent OTS. Rehabilitation guidelines should be viewed as reference points, not strict rules; clinicians are responsible for individualizing each rehabilitation pathway [18-25].

Known possible causes

As previously written in this article, there are no recognized causes, but rather a set of hypotheses. This chronic condition affects all body systems [38].

The age of the person could play a role in the onset of OTS, where advanced age would favor an easier detection of this syndrome, but the data are controversial [40,41]. Gender is another intrinsic factor, together with age, which would facilitate the onset of OTS. The female gender seems to be at a higher risk of overtraining compared to the male gender [40,42]. One explanation is the fluctuation of hormonal levels. Hormonal fluctuations would make the female gender more fatigued with the same volume of work compared to the male gender; this fatigue would make women more prone to having a longer time needed for physical recovery after an intense session, making the overcompensation process more fragile [41,42]. But there is no agreement in the literature with incontrovertible evidence. Recent research has highlighted that the hormonal level could fluctuate depending on the diet followed daily by the athlete, regardless of their gender [43].

According to some authors, it is the metabolic activity employed in training that determines which autonomic system is most negatively involved by inadequate recovery. Excessive commitment to aerobic or endurance training could cause a functional alteration of the parasympathetic system [36,37,44]. With a dysfunctional parasympathetic system, sympathetic tone is reduced, with bradycardia and accentuated chronic fatigue. In less recent studies evaluating OTS in endurance athletes, the tone of the vagal system decreased, with an increase in sympathetic tone [45,46].

An increase in sympathetic tone, according to some authors, occurs when OTS derives from intense training of anaerobic activities [36,37]. This non-physiological adaptation leads to alterations in cardiac rhythm and blood pressure, irritability, and insomnia. A recent study has highlighted that depending on the position of the subject studied, the sympathetic system reduces its activity in the sitting and lying positions, with a concomitant increase in parasympathetic tone. Conversely, in the upright position, the sympathetic system increases, and the vagal system is reduced [47]. The statement that the alteration of a specific autonomic system depends on the metabolism specifically employed in a given training is inadequate.

Another factor that could lead to OTS is the lack of neuroplasticity. According to some authors, the constant repetitiveness of the motor gesture would cause the creation of neural patterns to the detriment of the central and peripheral nervous system's ability to exploit its maximum capacity for adaptation. This would cause fragility in the nervous system when faced with demanding stress, with the onset of overtraining [48,49]. A recent study with an animal model has demonstrated that repetitiveness does not affect neuroplasticity [50]. Further studies are expected. OTS could reduce the ability to concentrate and reduce cognitive functions, in a reversible manner [51].

Another possible cause of OTS is chronically elevated levels of pro-inflammatory cytokines (and chemokines), such as tumor necrosis factor-alpha, interleukin-6, and C-reactive protein [52]. These cytokines prevent the muscles stimulated by training from recovering adequately, prolonging recovery times and leading to fatigue [52].

Another hypothesis linked to OTS is the inability of the muscles to exhaustively replace glycogen, leading to chronic fatigue [38]. A chronically elevated level of oxidative stress appears to be another factor in depleting a person's resources with linear training, causing muscle fatigue and a reduction in strength [53].

Chronic glutamine depletion could lead to chronic immune system dysfunction, with increased susceptibility to infections; this non-physiological environment could cause chronic fatigue and an inability to adequately recover resources between training sessions [54].

Some genetic polymorphisms could favor the onset of OTS, including COL5A1 and COL1A1 for collagen, altering muscle function and reducing its efficiency, with greater energy expenditure compared to adequate caloric intake [55]. This latter event could occur, causing the onset of OTS, by altering normal hormonal and immune function [40,56].

The presence of OTS increases the likelihood of falls and the onset of chronic diseases, both local (chronic inflammation) and systemic (cardiovascular system) [38-40].

Common features between COPD and OTS

There are common features between the presence of OTS (causes) and COPD. The multiplicity of symptoms and pathologies in these patients is known as multimorbidity [57].

In patients with COPD, we find collagen alterations in the structure of the airways, elevated oxidative stress, lower muscle glycogen levels compared to healthy subjects, glutamine depletion, impaired sympathetic nervous system function, chronic inflammation, osteoporosis, chronic fatigue, inadequate caloric intake, cardiovascular diseases, mood swings, and declining cognitive function [57-63].

Is the patient with COPD already on OTS? Is the patient with COPD more prone to OTS? These are unanswered questions, and further research is needed. Most likely, considering these data, it would be very appropriate to establish a rehabilitation program that includes sessions with reduced loads, rather than pursuing a linear increase in training work.

Much of the OTS literature cited derives from athletes, endurance training, or general sports physiology rather than chronic respiratory disease populations. This is not inherently inappropriate, as the analogy between athletes (who engage in physical activity) and patients (who engage in physical activity) exists. Naturally, further evidence and research are needed to obtain more evidence to support this relationship.

## Conclusions

The review stressed the need to consider rehabilitation training for patients with COPD not as a straight line upward, but as a sinusoidal line. Rehabilitation training must include a "wind-down" phase, where the amount of work is reduced, allowing the patient's body resources to be effectively recovered and performance to be improved to a greater extent. The presence of OTS risks delaying and/or eliminating the benefits deriving from the rehabilitation process. There are many symptoms and causes that OTS and COPD have in common, and further research is needed to optimally define the rehabilitation process.
